# Connection between Carbon Incorporation and Growth Rate for GaN Epitaxial Layers Prepared by OMVPE

**DOI:** 10.3390/ma12152455

**Published:** 2019-08-01

**Authors:** Timothy Ciarkowski, Noah Allen, Eric Carlson, Robert McCarthy, Chris Youtsey, Jingshan Wang, Patrick Fay, Jinqiao Xie, Louis Guido

**Affiliations:** 1 Department of Materials Science and Engineering, Virginia Tech, Blacksburg, VA 24061, USA; 2 Bradley Department of Electrical and Computer Engineering, Virginia Tech, Blacksburg, VA 24061, USA; 3 MicroLink Devices, Niles, IL 60714, USA; 4 Department of Electrical Engineering, University of Notre Dame, IN 46556, USA; 5 Qorvo, Inc., Richardson, TX 75080, USA

**Keywords:** III-nitride semiconductors, GaN-on-GaN homoepitaxy, organometallic vapor phase epitaxy, metal–organic chemical vapor deposition, carbon incorporation, electronic compensation, materials characterization, secondary ion mass spectroscopy

## Abstract

Carbon, a compensator in GaN, is an inherent part of the organometallic vapor phase epitaxy (OMVPE) environment due to the use of organometallic sources. In this study, the impact of growth conditions are explored on the incorporation of carbon in GaN prepared via OMVPE on pseudo-bulk GaN wafers (in several cases, identical growths were performed on GaN-on-Al_2_O_3_ templates for comparison purposes). Growth conditions with different growth efficiencies but identical ammonia molar flows, when normalized for growth rate, resulted in identical carbon incorporation. It is concluded that only trimethylgallium which contributes to growth of the GaN layer contributes to carbon incorporation. Carbon incorporation was found to decrease proportionally with increasing ammonia molar flow, when normalized for growth rate. Ammonia molar flow divided by growth rate is proposed as a reactor independent predictor of carbon incorporation as opposed to the often-reported input V/III ratio. A low carbon concentration of 7.3 × 10^14^ atoms/cm^3^ (prepared at a growth rate of 0.57 µm/h) was obtained by optimizing growth conditions for GaN grown on pseudo-bulk GaN substrates.

## 1. Introduction

Carbon has been shown by density functional theory to sit on the nitrogen sub-lattice and to behave as a compensator in n-type GaN [1,2]. Compensation in GaN epitaxial layers decreases electrical conductivity [3] which, in turn, increases on-resistance in high-power devices [4]. In addition, carbon has a detrimental effect on high-power device performance [5]. Drift layers in power devices with high blocking voltages require low n-type doping [6], (N_d_−N_a_) ≈ 1.5 × 10^15^ cm^−3^ for breakdown voltages approaching 20 kV. Controllability of free electron concentration at these low levels will be strongly influenced by carbon contamination.

Carbon is part of the organometallic molecule used as the gallium source Ga(CH_3_)_3_ in GaN growth via organometallic vapor phase epitaxy (OMVPE). Regardless of source purity and possible reduction of contamination from reactor parts, carbon will always be part of the OMVPE environment. The growth rate of GaN is limited by the availability of trimethylgallium (TMGa) due to the fact that most growth conditions have an ammonia (NH_3_) to TMGa ratio >> 1. Therefore, reducing the primary source of carbon, TMGa, would have a detrimental effect on the growth rate. Determining how to suppress carbon incorporation while minimizing the impact on growth rate would be desirable from the perspective of OMVPE throughput in an industrial setting.

In this study, growth conditions have been varied in order to learn more about the nature of carbon incorporation in GaN during the OMVPE process. In one case, the carrier gas velocity was increased and its mixture was changed to increase the growth efficiency. In the other case, the input NH_3_-to-TMGa (V/III) ratio was increased so as to enhance competition from nitrogen relative to carbon for sites on the anion sub-lattice. Other studies exist in which carbon incorporation was investigated as a function of OMVPE parameters [7,8,9,10]. However, in this prior work, carbon contamination exceeds mid 10^16^ atoms/cm^3^, which is almost two orders of magnitude higher than values found in our best GaN layers.

Growth efficiency (GE) is defined as the change in growth rate divided by the change in TMGa molar flow when the relationship between these two quantities is linear (i.e., when growth is limited by mass transport of TMGa to the gas-solid interface). OMVPE of III-N semiconductors has poor GE compared to other III–V compounds owing to strong parasitic reactions between NH_3_ and TMGa. Given this situation, it seems more appropriate to use growth rate (GR) instead of TMGa molar flow as a means of tracking and/or comparing carbon incorporation.

In the present work, carbon contamination in GaN of 3 × 10^15^ atoms/cm^3^ has been demonstrated at a GR of 2 µm/h. Moreover, a carbon level in GaN as low as 7.3 × 10^14^ atoms/cm^3^ has been achieved at a GR of 0.57 microns per hour. The connection between growth rate and residual carbon is not especially important for devices requiring only a few microns of material, but it is a significant consideration when trying to make 5–20 kV GaN power devices with 30–120 µm-thick drift layers.

## 2. Materials and Methods 

GaN epitaxial layers were grown in an AIXTRON 200/4 RF-S single wafer reactor with a quartz plate separating the line carrying organometallic (OM) sources from the line containing hydride (HYD) sources. The separation plate prevents mixing of OM and HYD sources before reaching the growth surface to assist in mitigating parasitic reactions. A calibrated optical pyrometer system located below a SiC-coated graphite susceptor was used to determine the growth temperature. TMGa was the source of gallium and NH_3_ was used as the source of nitrogen. Palladium-purified hydrogen was used as the carrier gas. The intentional source of silicon was 20 ppm SiH_4_ diluted in ultra-pure hydrogen.

Secondary ion mass spectroscopy (SIMS) analysis was performed by EAG Laboratories using conditions optimized for carbon sensitivity. Reported values of carbon concentration are taken as averages throughout layer thicknesses with the exclusion of interfacial spikes and dips due to changing TMGa flows.

The background carbon level found in the SIMS instrument was 1 × 10^15^ atoms/cm^3^. Therefore, to determine the carbon content in GaN layers, this background level must be subtracted from the raw measured (depth averaged) concentrations. This procedure was followed for all carbon concentration values reported herein. For example, the carbon concentration of 7.3 × 10^14^ atoms/cm^3^ was obtained by subtracting the background level of 1 × 10^15^ atoms/cm^3^ from the raw measured signal of 1.73 × 10^15^ atoms/cm^3^.

Epitaxial layers were grown mostly on pseudo-bulk GaN wafers prepared via hydride vapor phase epitaxy (HVPE); however, in several cases, identical growths were performed on GaN-on-Al_2_O_3_ templates made by OMVPE for comparison purposes. Carbon levels extracted from SIMS analysis were found to be nearly identical for growths done on the two substrates. The literature is inconclusive on this matter, with one study showing lower carbon and oxygen levels [11] and another study showing lower carbon but identical silicon levels [12] on pseudo-bulk GaN wafers compared to GaN-on-Al_2_O_3_ templates. In both cases, lower residual impurity content was attributed to reduced dislocation density in bulk GaN wafers. Growth rates were determined by measuring the depth of SIMS sputter craters and were found to be in excellent agreement for the two substrates.

**Figure 1:**
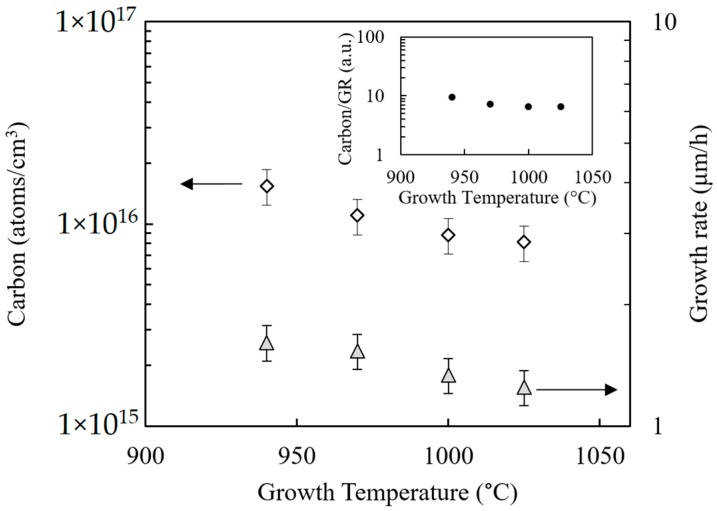
Carbon levels and GaN growth rates determined by Secondary ion mass spectroscopy (SIMS) analysis as a function of growth temperature for a test sample grown under Condition A. Inset: carbon concentration normalized by growth rate vs. growth temperature.

Growth temperature was selected by performing an experiment to establish the temperature above which the reaction of methyl groups from TMGa molecules appears to be complete [13]. The reactor pressure was fixed at 200 mbar and the TMGa flow was set to 6.26 × 10^−5^ mol/min. A GaN test sample was prepared containing four layers grown at different temperatures, and SIMS analysis was performed to determine the carbon concentration and growth rate for each layer. Figure 1 shows that the carbon concentration is almost constant with temperature above 975 °C, particularly if the carbon level is normalized by the growth rate (see inset). 

Given this result, the baseline condition for growth of high-quality GaN layers was selected to be a wafer temperature of 1000 °C, reactor pressure of 200 mbar, and TMGa flow of 6.26 × 10^−5^ mol/min. This set of parameters, along with gas flows and ratios listed in Table 1, is referred to hereafter as Condition A. Multiple Si-doped, 2 µm-thick GaN layers were grown under Condition A on GaN-on-Al_2_O_3_ templates to qualify the baseline material. Electron mobilities (obtained from Hall-effect measurements at room-temperature) were found to be in the range 600–700 cm^2^/Vs, with corresponding electron densities of 1–2 × 10^17^ electrons/cm^3^. These mobility values are consistent with high-quality Si-doped GaN layers grown on GaN-on-Al_2_O_3_ templates [14].

Two additional OMVPE conditions were implemented in this study using the same temperature and pressure set points as Condition A. The total carrier gas flow was increased in Condition B to make it possible to increase NH_3_ flow in Condition C. Then, the growth efficiency in Condition B was increased relative to Condition A by adjusting the flow ratios between the OM and HYD gas lines. Condition C has 4× higher NH_3_ flow relative to Condition B but the same total carrier gas flow and mixture. All three conditions resulted in uniform layer thicknesses and electrical properties across two-inch diameter wafers.

Epitaxial layer stacks for SIMS analysis, in which the TMGa flow was varied in four different regions, were made under each growth condition. TMGa flow was the chosen variable because of its importance as the source of the growth-limiting species, gallium, and the source of electrical compensation, carbon. In each region of constant TMGa flow, one half was un-intentionally doped (UID) and the other half was intentionally doped with silicon. The silane molar flow was held constant in all intentionally doped sections, but the actual silicon chemical content in both the Si-doped and UID sections varied with TMGa flow and growth conditions. The carbon concentration varied with TMGa flow but was identical in the UID and Si-doped sections. Two samples were grown for SIMS analysis using Condition C in order to evaluate reproducibility. Each SIMS chemical data point is given an error bar of ± 20%.

**Figure 2:**
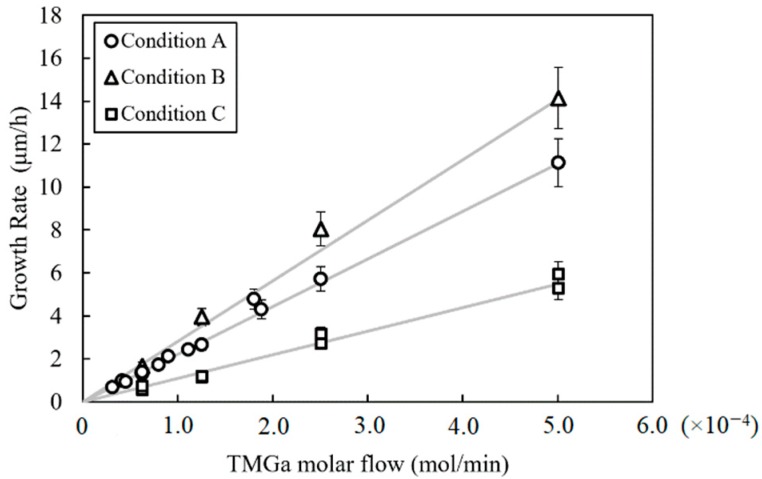
GaN growth rate vs. TMGa molar flow data (points) and curve fits (lines) for OMVPE conditions A, B, and C. Error bars are set to ±10%.

The linear relationships between GR and TMGa flow observed in Figure 2 establish that growth was mass transport limited and well behaved over a wide range of growth rates for all three conditions. Each growth condition in this study has a different GE (see Table 1), which is manifested in Figure 2 by different slopes for the curve fits. This indicates that a different amount of gallium reaches the growth surface and is incorporated for each growth condition at a given TMGa flow.

## 3. Results and Discussion

**Figure 3:**
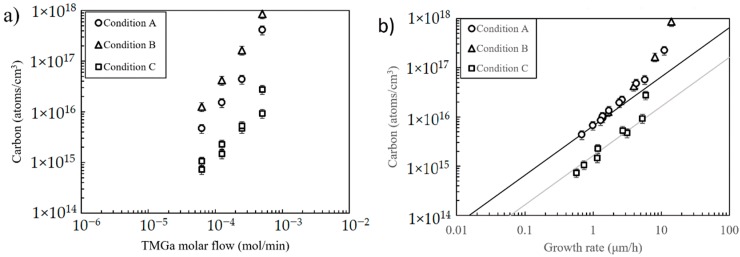
Carbon incorporation from SIMS analysis for all three OMVPE conditions plotted in (**a**) versus TMGa molar flow and in (**b**) versus growth rate.

Carbon contamination should increase with TMGa flow, as seen in Figure 3a, because TMGa is the “initial” (or input) source of both carbon and gallium and these two species do not compete for the same lattice sites (Ga→cation, C→anion). It is also evident in Figure 3a that carbon content at a fixed TMGa flow increases with growth efficiency (Condition B > A > C). These observations suggest the molecules that deliver Ga to the surface have arrival rates and sticking coefficients similar to those responsible for bringing C to the surface. In other words, carbon incorporation increases in proportion to its presence at the growth surface, which can be influenced either by changing the input TMGa flow or by improving the OMVPE process efficiency.

Carbon incorporation vs. GaN growth rate is plotted in Figure 3b, where it can be seen that data from Conditions A and B overlap. Moreover, the curve fit to this data is a power law with unity exponent (up to a growth rate of about 5 µm/h). This behavior suggests that only TMGa molecules (or other derivative molecules) contributing directly to GaN growth supply carbon for incorporation in the epitaxial layer. Moreover, it calls into question the conclusions from growth studies attempting to reduce carbon levels by “optimizing” OMVPE parameters without specifying the impact on the growth rate.

Interestingly, carbon incorporation becomes super-linear at growth rates higher than about 5 µm/h (Conditions A and B in Figure 3b). One possible reason for the sharp rise in carbon is incomplete conversion of methyl groups into more stable hydrocarbons [15]. Even if the relevant molecular conversion efficiencies remain the same, increasing TMGa flow (and thus growth rate) should increase the partial pressure of methyl groups—thereby increasing the availability of carbon in the gas phase.

The ammonia molar flow for Condition C is 4× higher than that used for Conditions A and B. Figure 3a shows Condition C results in the lowest values of carbon over the entire range of TMGa flows. This reduction in the carbon level is attributable to both the increased presence of NH_3_ molecules and the decrease in growth efficiency. It should be possible to adjust for the impact of lower GE alone by plotting carbon concentration vs. GaN growth rate (instead of TMGa flow). Note that in Figure 3b, carbon data from Condition C (grey trend line) still does not come into alignment with that from Conditions A and B (black trend line). That is, even after correcting for the smaller GE associated with Condition C, there remains about 4× reduction in carbon (relative to Conditions A and B) over a range of GR from 0.5 to 5 µm/h.

One explanation for this behavior is that enhanced chemical reaction between NH_3_ and TMGa (owing to 4× higher ammonia content) binds carbon into a molecule stable enough to withstand typical OMVPE temperatures without substantial pyrolysis. Another possibility is increased competition from nitrogen (owing to 4× higher NH_3_) relative to carbon (from TMGa) for atomic bonding sites on the anion sub-lattice. Moreover, if growth conditions are sufficient to convert all the methyl groups into hydrocarbons with a lower carbon incorporation efficiency [15] then, there would be even less competition from carbon for anion sub-lattice sites.

**Figure 4:**
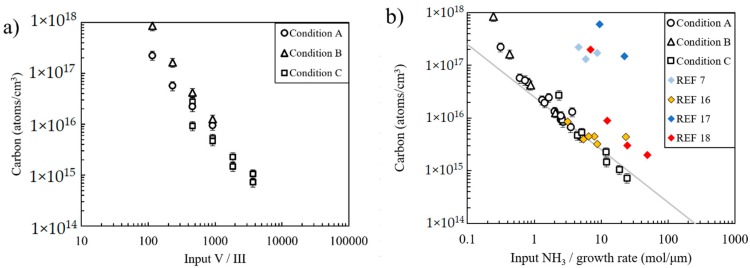
(**a**) Carbon incorporation versus input V/III ratio showing only data from this study. (**b**) Carbon incorporation versus input NH_3_/growth rate showing data from this study and data from the literature. The grey line is a guide to the eye aligned only to the data from this study, and it takes the form of a power law function with slope of −1.

Studies in the literature often plot carbon concentration versus input V/III ratio [16,17,18]. This type of plot is presented in Figure 4a for the samples considered in this study. Overall, there is a clear trend of decreasing carbon with increasing input V/III ratio; however, data are spread out along the carbon concentration axis because of differences in GE for the three growth conditions (A, B, C).

Plotting carbon content vs. input V/III ratio takes into account the competition between carbon and nitrogen for anion lattice sites. However, plotting carbon vs. input NH_3_/growth rate, as shown in Figure 4b, also takes into account the different growth efficiencies. Note the much tighter grouping of carbon concentration values in Figure 4b for samples considered in this study. Also included in Figure 4b are data from the literature [7,16,17,18], for cases in which input NH_3_ molar flow and growth rate were reported. The data from Piao et al. [16] are in good agreement with findings from the present work, despite differences in reactor design and reactor pressure (atmospheric vs. 200 mbar). The other data [7,17,18] fall well above the trend line, suggesting higher carbon incorporation efficiency. It appears that the trend line found in the present study sets a lower bound on carbon incorporation over a wide range of input NH_3_/growth rate settings.

## 4. Conclusions

For GaN epitaxial layers in this study, TMGa is both the source of gallium and the source of residual carbon. It was found that only TMGa contributing to the growth rate contributes to carbon incorporation. The work reported here shows that input NH_3_/growth rate is more appropriate than input V/III ratio for tracking carbon incorporation because the former takes into account the growth efficiency of the OMVPE process. A carbon concentration of 7.3 × 10^14^ atoms/cm^3^ was found in the present work for a growth rate of 0.57 μm/h. This carbon contamination level is the lowest reported in the literature for OMVPE-grown GaN.

## Figures and Tables

**Table 1:** OMVPE parameters associated with growth conditions A, B, and C.

Growth Conditions	NH_3_ Molar Flow (mol/min)	Total Gas Flow (mol/min)	HYD/OM Flow Ratio	Growth Efficiency (µm/mol)
Condition A	0.06	0.18	0.98	369
Condition B	0.06	0.28	5.36	469
Condition C	0.23	0.28	5.36	183

OMVPE, organometallic vapor phase epitaxy; HYD/OM: hydride /organometallic.
